# Testis- and ovary-expressed polo-like kinase transcripts and gene duplications affect male fertility when expressed in the *Drosophila melanogaster* germline

**DOI:** 10.1093/g3journal/jkae273

**Published:** 2024-11-20

**Authors:** Paola Najera, Olivia A Dratler, Alexander B Mai, Miguel Elizarraras, Rahul Vanchinathan, Christopher A Gonzales, Richard P Meisel

**Affiliations:** Department of Biology and Biochemistry, University of Houston, Houston, TX 77204, USA; Department of Biology and Biochemistry, University of Houston, Houston, TX 77204, USA; Department of Biology and Biochemistry, University of Houston, Houston, TX 77204, USA; Department of Biology and Biochemistry, University of Houston, Houston, TX 77204, USA; Department of Biology and Biochemistry, University of Houston, Houston, TX 77204, USA; Department of Biology and Biochemistry, University of Houston, Houston, TX 77204, USA; Department of Biology and Biochemistry, University of Houston, Houston, TX 77204, USA

**Keywords:** spermatogenesis, testis, nondisjunction, sexual antagonism, meiosis

## Abstract

Polo-like kinases (Plks) are essential for spindle attachment to the kinetochore during prophase and the subsequent dissociation after anaphase in both mitosis and meiosis. There are structural differences in the spindle apparatus among mitosis, male meiosis, and female meiosis. It is therefore possible that alleles of Plk genes could improve kinetochore attachment or dissociation in spermatogenesis or oogenesis, but not both. These opposing effects could result in sexually antagonistic selection at Plk loci. In addition, Plk genes have been independently duplicated in many different evolutionary lineages within animals. This raises the possibility that Plk gene duplication may resolve sexual conflicts over mitotic and meiotic functions. We investigated this hypothesis by comparing the evolution, gene expression, and functional effects of the single Plk gene in *Drosophila melanogaster* (*polo*) and the duplicated Plks in *D. pseudoobscura* (*Dpse-polo* and *Dpse-polo-dup1*). *Dpse-polo-dup1* is expressed primarily in testis, while other *Drosophila* Plk genes have broader expression profiles. We found that the protein-coding sequence of *Dpse-polo-dup1* is evolving significantly faster than a canonical *polo* gene across all functional domains, yet the essential structure of the encoded protein has been retained. We present additional evidence that the faster evolution of *Dpse-polo-dup1* is driven by the adaptive fixation of amino acid substitutions. We also found that over or ectopic expression of *polo* or *Dpse-polo* in the *D. melanogaster* male germline resulted in greater male infertility than expression of *Dpse-polo-dup1*. Last, expression of *Dpse-polo* or an ovary-derived transcript of *polo* in the male germline caused males to sire female-biased broods, suggesting that some Plk transcripts can affect the meiotic transmission of the sex chromosomes in the male germline. However, there was no sex bias in the progeny when *Dpse-polo-dup1* was ectopically expressed, or a testis-derived transcript of *polo* was overexpressed in the *D. melanogaster* male germline. Our results therefore suggest that *Dpse-polo-dup1* may have experienced positive selection to improve its regulation of the male meiotic spindle, resolving sexual conflict over meiotic Plk functions. Alternatively, *Dpse-polo-dup1* may encode a hypomorphic Plk that has reduced deleterious effects when overexpressed in the male germline. Similarly, testis transcripts of *D. melanogaster polo* may be optimized for regulating the male meiotic spindle, and we provide evidence that the untranslated regions of the *polo* transcript may be involved in sex-specific germline functions.

## Introduction

Gametogenesis in animals is sexually dimorphic. Sex differences in gametogenesis start with the establishment of the germline, continue through meiosis, and conclude with sexually dimorphic sperm and eggs (Fuller and Spradling 2007; Whitworth *et al*. 2012; Lehtonen *et al*. 2016; Cahoon and Libuda 2019). Meiosis, a central process of gametogenesis, is highly differentiated between the sexes (Hua and Liu 2021). Male meiosis starts with a single diploid cell and produces four haploid sperm; in contrast, female meiosis produces a single haploid egg and two polar bodies from a diploid precursor (Evans and Robinson 2011; McKee *et al*. 2012). There are additional sex differences in the meiotic spindle apparatus, meiotic chromatin, chromosomal pairing, and recombination rates (Orr-Weaver 1995; McKee 1996; Sardell and Kirkpatrick 2020).

Intersexual differences in gametogenesis create numerous opportunities for intragenomic and intersexual conflicts (Arnqvist and Rowe 2013; Rice 2013). For example, one allele of a gene may improve some aspect of spermatogenesis, while negatively affecting oogenesis, and vice versa for the alternative allele (VanKuren and Long 2018; Hamada *et al*. 2020). This type of intralocus intersexual conflict (or sexual antagonism) may be resolved by gene duplication, followed by specialization (or subfunctionalization) of one copy for spermatogenesis or gametogenesis (Tracy *et al*. 2010; Connallon and Clark 2011; Gallach and Betrán 2011). Such germline-specific sexual subfunctionalization may be common for genes involved in sex-specific or sexually dimorphic aspects of meiosis (Reis *et al*. 2011).

Intersexual conflicts likely arise because of differences among the mitotic, female meiotic, and male meiotic spindle apparatus (Orr-Weaver 1995; Savoian and Glover 2014). Despite the differences across mitotic and meiotic spindles, many genes encode proteins that are required for the mitotic, female meiotic, and male meiotic spindles. For example, the *Drosophila melanogaster* gene *mad2* encodes a protein involved in the mitotic and meiotic spindle assembly checkpoints (Li and Murray 1991; Shah and Cleveland 2000; Nicklas *et al*. 2001; Tsuchiya *et al*. 2011). In the lineage leading to *D. pseudoobscura*, *mad2* was duplicated, and each copy may have evolved a specialized meiotic function in either males or females (Meisel *et al*. 2010). It is possible that sex-specific subfunctionalization of each paralog resolved an intersexual conflict that arose because of sexually dimorphic meiotic spindles. However, there has yet to be a direct test of the hypothesis that sex differences in the meiotic spindle create sexual antagonism.

Here, we use the *Drosophila* gene *polo* as a model to explore intersexual conflicts that arise as a result of the sexually dimorphic meiotic spindle aparatus. Polo-like kinases (Plks) are essential regulators of both mitosis and meiosis across eukaryotes (Archambault and Glover 2009). Specifically, Plks are required for spindle attachment to the kinetochore during prophase and the subsequent dissociation of the kinetochore after anaphase (Sunkel and Glover 1988; Llamazares *et al*. 1991; Donaldson *et al*. 2001). The *D. melanogaster* genome has a single Plk gene (*polo*), which is necessary for chromosome segregation during meiosis in both oogenesis and spermatogenesis (Sunkel and Glover 1988; Carmena *et al*. 1998; Herrmann *et al*. 1998; Das *et al*. 2016). Loss of function *polo* mutations affects oogenesis and early embryogenesis—from oocyte determination through meiosis and into the establishment of the embryonic sperm aster (Sunkel and Glover 1988; Tavares *et al*. 1996; Riparbelli *et al*. 2000; Mirouse *et al*. 2006). Polo is similarly required for meiotic chromosome segregation during spermatogenesis; males with *polo* mutations experience high rates of nondisjunction and produce sperm with abnormal DNA content, likely because Polo is involved in the attachment of kinetochores to the spindle apparatus (Sunkel and Glover 1988; Carmena *et al*. 1998, 2014; Herrmann *et al*. 1998). Given the differences in meiotic spindles between male and female *Drosophila* (Orr-Weaver 1995), it is possible that *polo* alleles may have sexually antagonistic effects if they improve kinetochore attachment and dissolution in spermatogenesis or oogenesis, but not both.

We evaluated whether *polo* has sexually antagonistic effects in *Drosophila*, and we also explored whether that conflict was subsequently resolved by testis-specific specialization of a *polo* gene duplication. While *D. melanogaster* has a single Plk gene (*polo*), the *D. pseudoobscura* genome harbors two duplications (three total copies) of *polo* (Reis *et al*. 2011). *D. melanogaster polo* is autosomal (on chromosome *3L* or *Drosophila* Muller element D), and the chromosome carrying *polo* fused to the *X* chromosome in the lineage leading to *D. pseudoobscura.* Therefore, the *D. pseudoobscura* ortholog of *polo* (*Dpse-polo*) is on a neo*-X* chromosome. An excess of genes was duplicated from the *D. pseudoobscura* neo-*X* chromosome to the autosomes (Meisel *et al*. 2009), including *polo* (Reis *et al*. 2011). The two duplicate copies of *polo* (*polo-dup1* and *polo-dup2*) are expressed primarily in males in *D. persimilis* (the sibling species of *D. pseudoobscura*), while the ancestral copy of *polo* is expressed in both sexes (Reis *et al*. 2011). The divergence in expression between *polo* paralogs is consistent with sex-specific subfunctionalization of a duplicated gene to resolve an intersexual conflict (Gallach and Betrán 2011). Only *polo-dup1* is predicted to encode a complete Plk, suggesting that *polo-dup1* may have been retained to resolve an intersexual conflict, while *polo-dup2* may be a pseudogene. We examined the expression and evolution of the ancestral *D. pseudoobscura polo* (*Dpse-polo*) and the complete duplication (*Dpse-polo-dup1*). We also cloned Plk transcripts into vectors for the GAL4>UAS binary expression system, and we tested whether expressing these different *polo* transgenes in the male germline affects male fertility and the sex ratio of progeny sired by these males.

## Materials and methods

### Plk expression

We compared the transcribed regions of *D. melanogaster polo*, *Dpse-polo*, and *Dpse-polo-dup1* in testes and ovaries. For *D. melanogaster*, we obtained RNA-seq read mapping coverage data for testes and ovaries (both virgin and mated females) from the FlyBase JBrowse representation of modENCODE RNA-seq data (Brown *et al*. 2014; Buels *et al*. 2016; Öztürk-Çolak *et al*. 2024). For *D. pseudoobscura*, we obtained RNA-seq read mapping coverage data for testes and ovaries from the Genomics Education Partnership mirror of the UCSC Genome Browser (Yang *et al*. 2018; Rele *et al*. 2022).

We also compared the expression of *Dpse-polo* and *Dpse-polo-dup1* in males and females across *D. pseudoobscura* tissue samples. We first obtained normalized read counts (NRCs) for all *D. pseudoobscura* genes from an RNA-seq data set in which expression was measured in four replicates from each sex for seven different tissue samples (GSE99574; Yang *et al*. 2018). NRC data are available in Supplementary File 1. We calculated the median NRC for each gene across all four replicates for each tissue-by-sex combination (NRC_TS_), and then we analyzed log_10_(NRC_TS_ + 1). We added one to each NRC_TS_ value to ensure that all values were finite (because some NRC_TS_ values were equal to zero). We compared log_10_(NRC_TS_ + 1) of *Dpse-polo* (FBgn0071596) and *Dpse-polo-dup1* (FBgn0246554) to the genome-wide distribution of log_10_(NRC_TS_ + 1) values to evaluate the relative expression of each *polo* gene in teach tissue-by-sex combination. R Code to perform this analysis is available in Supplementary File 2.

We used the same RNA-seq data to calculate the breadth of expression (τ) across six nonoverlapping tissue samples for *Dpse-polo* and *Dpse-polo-dup1*: (1) digestive plus excretory system, (2) gonad, (3) reproductive system without gonad, (4) thorax without digestive system, (5) abdomen without digestive or reproductive system, and (6) head. We calculated τ with the following equation (Yanai *et al*. 2005):


$$\tau =\frac{\left(\sum_{i=1}^{N}\;1-\frac{\mathrm{lo}g_{10}\;(S_{i}+1)}{\mathrm{lo}g_{10}\;(S_{\max}+1)}\right)}{\left(N-1\right)}.$$

In this equation, expression of a gene in *N* = 6 tissues is measured as log_10_(*S_i_* +1), where *S_i_* is the NRC_TS_ in tissue *i* for a given sex. *S*_max_ is the maximum *S_i_* of the gene across all six tissue samples in a given sex. Values of τ range from 0 (equal expression in all tissues, i.e. broadly expressed) to 1 (expressed in a single tissue, i.e. narrowly expressed). We calculated τ separately for male and female tissue samples. R Code to perform this analysis is available in Supplementary File 2.

### Evolution of Plk protein sequences

We tested for differences in the rates of evolution of the amino acid sequences encoded by *Dpse-polo* and *Dpse-polo-dup1*. A previous analysis found that the nucleotide sequence of *Dpse-polo-dup1* evolves faster than *Dpse-polo* (Reis *et al*. 2011), but the rate of amino acid evolution was not directly examined. To address that shortcoming, we constructed an amino acid alignment of Dpse-Polo (XM_001353282), Dpse-Polo-dup1 (XM_002132425), and *D. melanogaster* Polo (FBtr0074839) using MUSCLE implemented in MEGA 11 for macOS with the default parameters (Edgar 2004; Stecher *et al*. 2020; Tamura *et al*. 2021). The alignment is available as Supplementary File 3. We then used Tajima's (1993) relative rate test to compare the number of amino acid substitutions in the evolutionary lineages leading to Dpse-Polo and Dpse-Polo-dup1, treating *D. melanogaster* Polo as the outgroup. We analyzed amino acid substitutions only because synonymous substitutions are saturated between *D. melanogaster* and *D. pseudoobscura* (Richards *et al*. 2005). We also compared the number of amino acid substitutions within the N-terminal serine/threonine kinase domain, the Polo box domain (PBD), the two individual Polo boxes (PB1 and PB2), and the linker between the kinase domain and PBD.

We additionally performed a McDonald–Kreitman (MK) test (McDonald and Kreitman 1991) to determine whether there were an excess of nonsynonymous substitutions within either *Dpse-polo* (GA11545) or *Dpse-polo-dup1* (GA25172). To do so, we first obtained aligned protein-coding sequences for each gene from 30–31 strains of *D. pseudoobscura* and 11 strains of *D. miranda* (a close relative) from PseudoBase (Korunes *et al*. 2021). We then used the aligned sequences (Supplementary Files 4 and 5) to compare the number of nonsynonymous and synonymous polymorphic sites within each species and fixed differences between species with DnaSP v6 (Rozas *et al*. 2017).

### Creating transgenic *D. melanogaster* carrying inducible Plk transcripts

We cloned Plk transcripts from *D. melanogaster* testes, *D. melanogaster* ovaries, and whole *D. pseudoobscura* males. *D. melanogaster* testis and ovary tissues were dissected in Ringer's solution from whole flies of the iso-1 strain (BDSC 2057). Ovaries and testes were dissolved overnight in TRI Reagent on a rocker. Whole *D. pseudoobscura* males (from the MV2-25 strain) were ground in TRI Reagent with a motorized pestle and centrifuged to remove particulates. We used the Direct-zol RNA Purification Kit (Zymo Research) to isolate RNA from each sample, following the manufacturer's instructions.

**Fig. 1.**
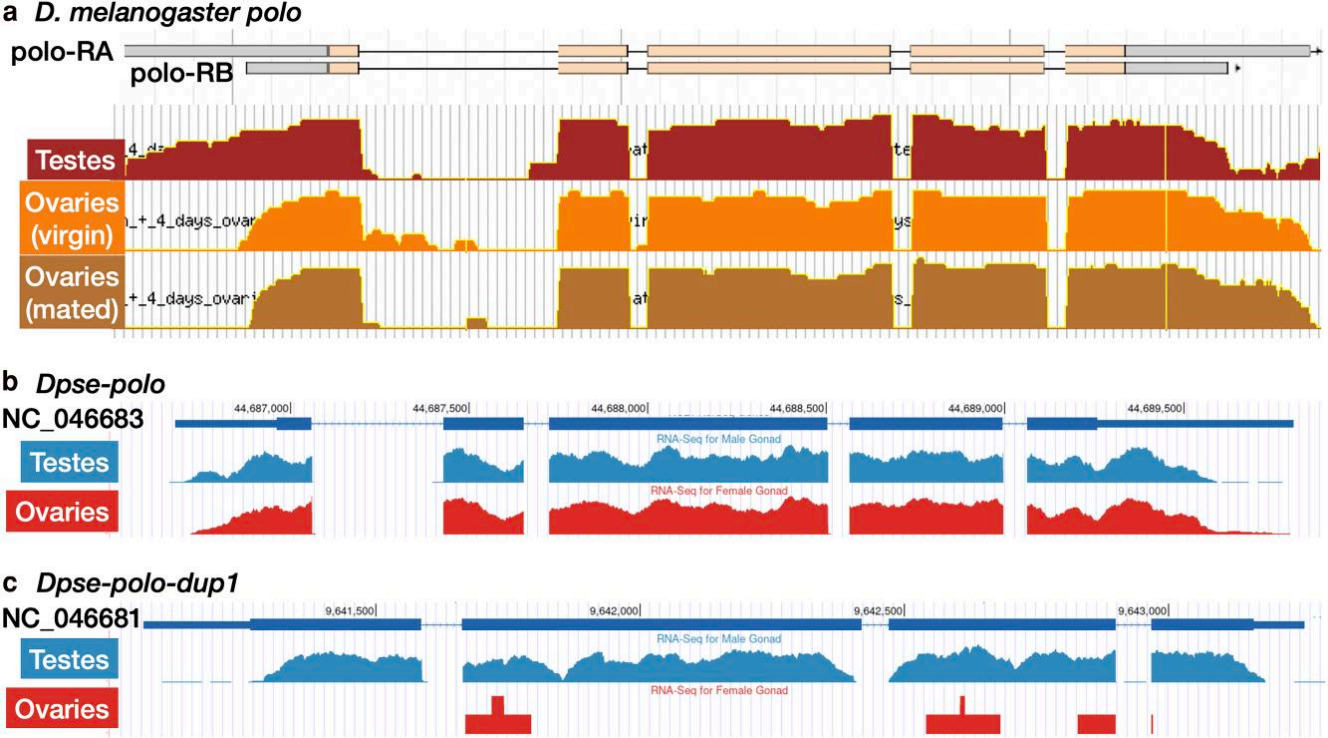
RNA-seq read mapping across the *polo*, *Dpse-polo*, and *Dpse-polo-dup1* gene regions. a) Transcript and exon structures of polo-RA and polo-RB are shown, with UTRs in gray and protein-coding sequence in tan. RNA-seq reads mapped per nucleotide position are shown for samples from testes, ovaries (virgin females), and ovaries (mated females). b and c) Transcript and exon structures of *Dpse-polo* and *Dpse-polo-dup1* are shown, with UTRs as thinner bars and protein-coding sequence as thicker bars. RNA-seq reads mapped per nucleotide position are shown for samples from testes and ovaries. Images downloaded and modified from a) FlyBase JBrowse or b and c) the GEP mirror of the UCSC Genome Browser.

The resultant RNA samples were used as templates in a reverse transcription PCR with primers targeting *polo* (*D. melanogaster* testis or ovary), *Dpse-polo* (*D. pseudoobscura* males), or *Dpse-polo-dup1* (*D. pseudoobscura* males) using SuperScript III reverse transcriptase (Thermo Fisher Scientific). Different primer pairs were used to amplify *polo* from *D. melanogaster* ovaries (poloO) and testes (poloT) because the primers for one tissue sample would not amplify the transcript from the other tissue sample. Each of the four cDNA products was then used as a template in a PCR with the same primers and Phusion High Fidelity DNA Polymerase (New England Biolabs). All primer pairs were located within the 5′- and 3′- untranslated regions (UTRs) of the transcripts, so that they amplified the entire protein-coding sequence of the respective genes (Fig. 1; Supplementary Table 1). A “CACC” adapter sequence was included at the 5′ end of each forward primer to allow the PCR products to be cloned into a Gateway Entry vector.

We used the Gateway System to clone each PCR product into a vector that could be used for germline transformation of *D. melanogaster*. We first used the pENTR/D-TOPO Cloning Kit to create Gateway Entry clones for each of the four PCR products, which we transformed into One Shot TOP10 Chemically Competent *Escherichia coli* cells (Thermo Fisher Scientific). We then isolated plasmids from all four cloning products with the Invitrogen PureLink Quick Plasmid Miniprep kit. We confirmed the correct insert size using PCR with the M13 primer pair. We next used the Gateway LR Clonase II Enzyme mix to recombine each of the four PCR products into the pBID-UASC-G backbone (Addgene Plasmid #35202), which contains a ϕC31 integrase compatible attB sequence and UAS-binding sites for the GAL4 expression system (Wang *et al*. 2012). We transformed One Shot TOP10 Chemically Competent *E. coli* cells with each of the four recombinant plasmids. We designed primers to amplify the inserts within the pBID-UASC-G plasmid (5′-TGCCGCTGCCTTCGTTAATA-3′ and 5′-TTCCACCACTGCTCCCATTC-3′), and we confirmed that the inserts were the correct size. We also used Sanger sequencing of the PCR products to confirm that there were no DNA sequence errors in the resulting amplifications. We finally used the Invitrogen PureLink HiPure Plasmid Filter Midiprep Kit to isolate plasmids containing each of the four PCR products.

We created transgenic *D. melanogaster* that carry one of each of the four recombinant plasmids. Each of the four plasmids was injected into *D. melanogaster* strain VK20 (BDSC 9738), which has an attP docking site at region 99F8 of chromosome *3R*. All injections were performed by GenetiVision Corporation. We confirmed successful transformation via the presence of orange eyes. We balanced the third chromosome carrying each of the transgenes over a TM3, Sb chromosome. Each of these strains has the genotype UAS-poloX/TM3, Sb, where poloX refers to the specific Plk transcript (poloO, poloT, *Dpse-polo*, or *Dpse-polo-dup1*). We created at least one (and no more than three) balanced strains for each transgene, with each strain originating from a different transformed founder (Supplementary Table 2).

### Assaying effects of Plk transcripts on male fertility and progeny sex ratios

We tested whether male germline expression of each of the four Plk transcripts affects male fertility and sex chromosome transmission. Males with the UAS-poloX/TM3, Sb genotype were mated to females carrying a Gal4 driver construct that is expressed under the *bag of marbles* (*bam*) promoter (*P*{*bam-Gal4-VP16*}), which drives expression in the male germline (Chen and McKearin 2003; Sartain *et al*. 2011; Hart *et al*. 2018). After mating, all flies, eggs, and larvae were kept in 25 × 95 mm vials containing cornmeal media in 25°C incubators with 12:12 light:dark cycles. Male progeny with the *P*{*bam-Gal4-VP16*}>UAS-poloX genotype were identified by wild-type bristles.

We assayed male fertility by allowing *P*{*bam-Gal4-VP16*}>UAS-poloX males to mate with wild-type females from the Canton S (CanS) and Oregon R (OreR) strains. A single male and single female were combined in a 25 × 95 mm vial with cornmeal media at 25°C, and they were observed to confirm successful copulation, as we have done previously with crosses using the same *P*{*bam-Gal4-VP16*} strain (Hart *et al*. 2018). After mating, the male was removed from the vial, and the female was allowed to lay eggs for 3–5 days at 25°C. The vials were stored at 25°C, and we counted the number of male and female progeny that emerged in each vial for 21 days after mating.

We tested for an effect of germline expression of each Plk transgene on the number of progeny using mixed effect linear models. Our analysis compared the effects of UAS-poloO, UAS-poloT, UAS-*Dpse-polo*, and UAS-*Dpse-polo-dup1*. We analyzed all strains with the same transgene within a single model, treating strain as a random effect. For each comparison, we used the lme() function within the nlme package in R (Pinheiro and Bates 2000; Pinheiro *et al*. 2024) to construct a linear model with the number of progeny in a vial as a response variable, transgene as a fixed effect, and batch and strain as random effects (see Supplementary File 6 for R code). We tested for an effect of each transgene by separately analyzing the total number of progeny per vial, the number of male progeny, or the number of female progeny.

We also used mixed effects logistic regression to test if the transgenes affected whether a male sired any offspring. As above, we compared the effects of UAS-poloO, UAS-poloT, UAS-*Dpse-polo*, and UAS-*Dpse-polo-dup1*, including all strains with the same transgene in a single model. For each comparison, we performed a logistic regression using the glmer() function in the lme4 package (Bates *et al*. 2015) to construct a model with whether a male sired progeny as a response variable (0 = no, 1 = yes), transgene as a fixed effect, and batch and strain as random effects (see Supplementary File 6 for R code). We tested for an effect of each transgene by separately analyzing if any progeny were sired, if male progeny were sired, or if female progeny were sired.

We additionally tested for differences in the sex ratio (relative numbers of male and female progeny) using mixed effect linear models. As above, we analyzed all strains with the same transgene within a single model. For each transgene, we used the lme() function in the nlme package (Pinheiro and Bates 2000; Pinheiro *et al*. 2024) to construct a linear model with the number of progeny as response variable, progeny sex (male or female) and vial as fixed effects, and batch and strain as random effects (see Supplementary File 6 for R code). We conclude that a transgene affects the sex ratio when progeny sex has a significant effect on the number of progeny.

## Results

### Dpse-polo-dup1 is highly expressed in male reproductive tissues

We compared the expression of *D. melanogaster polo*, *Dpse-polo*, and *Dpse-polo-dup1* in testes and ovaries using available RNA-seq data (Brown *et al*. 2014; Yang *et al*. 2018). We first confirmed that *D. melanogaster polo* is expressed in both testes and ovaries (Fig. 1a). There are two annotated splice variants of *polo*, which differ in the length of their UTRs: polo-RA has longer 5′- and 3′-UTRs than polo-RB (Fig. 1a). Curiously, the testis and ovary transcripts of *polo* appear to have atypical UTR configurations that each differ from polo-RA and polo-RB (Fig. 1a). Specifically, RNA-seq reads from testis transcripts map to the longer 5′-UTR (similar to polo-RA) but not the longer 3′-UTR (similar to polo-RB). In contrast, RNA-seq reads from ovary transcripts map to the longer 3′-UTR (similar to polo-RA) but not the longer 5′-UTR (similar to polo-RB).

*Dpse-polo* is expressed in both testes and ovaries (Fig. 1b), similar to *D. melanogaster polo*. There is some evidence for a longer 5′-UTR in the testis transcripts from *Dpse-polo*, but the enrichment is not as prevalent as in *D. melanogaster polo* transcripts from testes. There does not appear to be a difference in the 3′-UTR of *Dpse-polo* between testis and ovary transcripts. In contrast to *polo* and *Dpse-polo*, *Dpse-polo-dup1* is predominantly expressed in testes, with very little evidence for expression in ovaries (Fig. 1c). This is consistent with the previously documented evidence of male-biased expression of *polo-dup1* in *D. persimilis* (Reis *et al*. 2011).

**Fig. 2.**
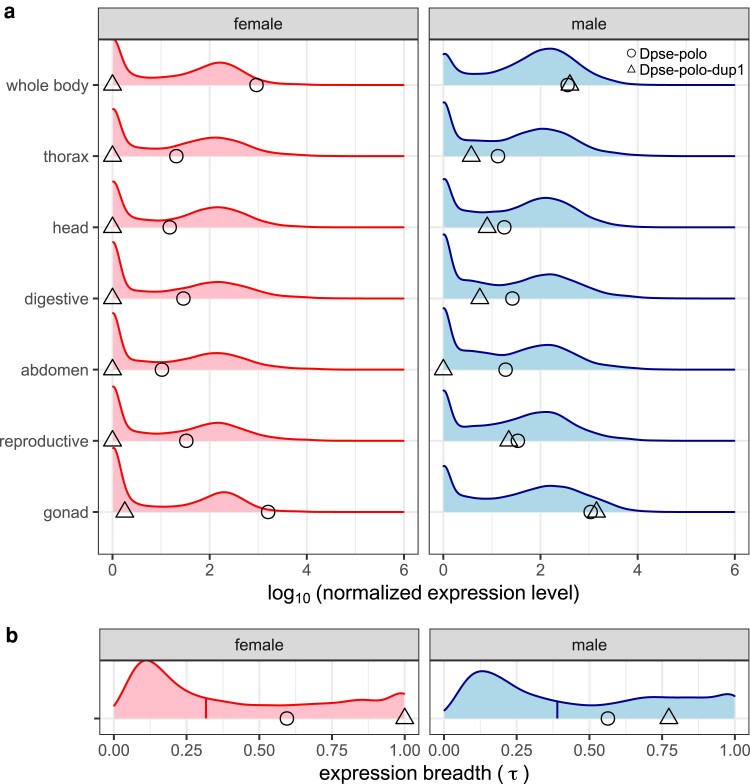
a) Expression of *Dpse-polo* and *Dpse-polo-dup1* across seven different tissue samples in males and females. The *X*-axis shows the log_10_ of the median normalized expression (GSE99574; Yang *et al*. 2018). Each distribution shows the expression level of all genes in a given sex-by-tissue combination. The circles show the expression of *Dpse-polo*, and the triangles show the expression level of *Dpse-polo-dup1* in each sample type. Tissue samples are whole body, thorax (with digestive system removed), head, digestive system (including excretory system), abdomen (with digestive and reproductive system removed), reproductive system (without gonad), and gonad (ovary or testis). b) The distribution of expression breadth (τ) of all genes across six unique tissue samples (excluding whole body) in females or males is plotted. The vertical line segments within each distribution show the median value. The circles show the expression breadth of *Dpse-polo*, and the triangles show the expression breadth of *Dpse-polo-dup1*.

We further tested whether *Dpse-polo-dup1* has male-biased expression by using available RNA-seq data to compare the expression of *Dpse-polo* and *Dpse-polo-dup1* across seven different tissue samples in both males and females (Fig. 2a). In each tissue sample, we observed a bimodal distribution of genome-wide expression levels, with one distribution centered close to zero (lowly expressed genes) and another distribution centered ∼2 orders of magnitude higher (highly expressed genes). In all sex-by-tissue combinations, *Dpse-polo* was expressed at a level within the distribution of highly expressed genes. In contrast, *Dpse-polo-dup1* was not expressed or expressed at a low level across all female tissue samples and most male samples. The notable exceptions were male samples that included reproductive tissues (whole body, reproductive system, and gonad), in which *Dpse-polo-dup1* was highly expressed, similar to *Dpse-polo*. The highest expression of *Dpse-polo-dup1* was in testis.

We quantified the expression breadth of *Dpse-polo* and *Dpse-polo-dup1* using τ, which ranges from 0 (equally expressed in all tissues) to 1 (only expressed in a single tissue). *Dpse-polo* had a similar expression breadth in both females (τ = 0.59) and males (τ = 0.56), which was larger than the median τ across the genome (Fig. 2b). The high τ of *Dpse-polo* could be attributed to elevated expression in gonads relative to other tissue samples, but *Dpse-polo* was highly expressed across all tissues (Fig. 2a). Surprisingly, *Dpse-polo-dup1* had the maximal τ value of 1 when expression was measured in females (Fig. 2b). This is because expression was only detected in the ovary, yet Dpse-polo-dup1 is expressed at a very low level in ovary (Figs. 1 and 2). In males, *Dpse-polo-dup1* had substantially more tissue-specific expression (τ = 0.77) than Dpse-polo, and this was caused by extremely high expression of Dpse-polo-dup1 in testis (Fig. 2). We therefore conclude that *Dpse-polo-dup1* has almost completely male-limited expression and strong testis-biased expression.

### Accelerated evolution of Dpse-polo-dup1

We compared the number of amino acid substitutions in *Dpse-polo* and *Dpse-polo-dup1* to test for accelerated evolution along the lineage leading to *Dpse-polo-dup1* (Supplementary Table 3). There were significantly more amino acid substitutions in the lineage leading to *Dpse-polo-dup1* than *Dpse-polo* ($\chi_{1}^{2}$ = 71.43, *P* < 0.00001), consistent with the previously described faster evolution in the nucleotide sequence of *Dpse-polo-dup1* (Reis *et al*. 2011). Of the 567 alignable amino acid positions, 80 residues (14%) were estimated to be divergent along the lineage leading to *Dpse-polo-dup1*. In contrast, only three amino acid substitutions were identified along the lineage leading to *Dpse-polo*.

**Fig. 3.**
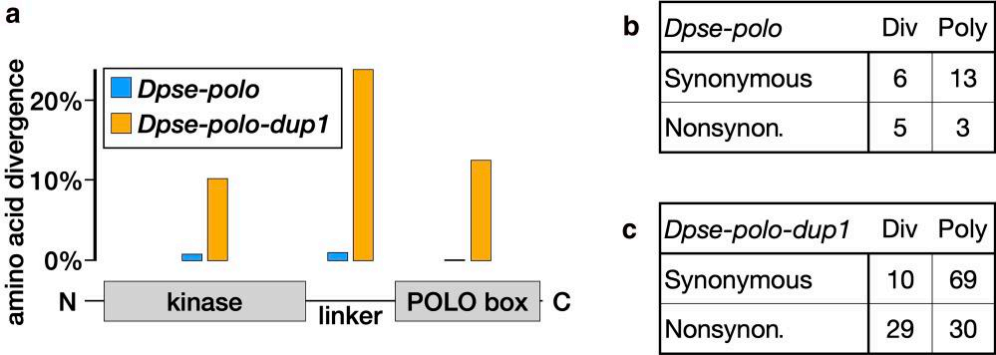
Accelerated protein-coding divergence in *Dpse-polo-dup1* relative to *Dpse-polo*. a) Each bar shows the percent of amino acids with a substitution along the lineage leading to either *Dpse-polo* or *Dpse-polo-dup1*, out of all alignable sites. Divergence was calculated within the N-terminal serine/threonine kinase domain (kinase), the C-terminal PBD, and the linker. Counts of amino acid substitutions are provided in Supplementary Table 3. b and c) Tables show the counts of synonymous and nonsynonymous (Nonsynon.) sites that are fixed differences between *D. pseudoobscura* and *D. miranda* (Div) or polymorphic within *D. pseudoobscura* (Poly) for *Dpse-polo* or *Dpse-polo-dup1*.

We next explored amino acid divergence along the lineage leading to *Dpse-polo* and *Dpse-polo-dup1* across the different domains of the Polo protein. Plks consist of an N-terminal serine/threonine kinase domain and a C-terminal Polo box domain (PBD), separated by a linker. Both the kinase domain and PBD are present without any insertions or deletions in both *Dpse-polo* and *Dpse-polo-dup1*. The PBD can be further divided into Polo box 1 (PB1) and Polo box 2 (PB2), and there are two amino acids (histidine at position 518 and lysine at position 520) that are required to bind Polo targets (Elia *et al*. 2003a, 2003b). Both residues are conserved in *Dpse-polo* and *Dpse-polo-dup1*. There were nine amino acids deleted in *Dpse-polo-dup1* (out of a total of 576 codons in *D. melanogaster polo*), and all 9 are located in the linker (Supplementary File 3). One of those amino acids was also deleted in *Dpse-polo*. Despite the structural conservation of *Dpse-polo-dup1*, there were significantly more amino acid substitutions in the kinase domain, PBD, and linker of *Dpse-polo-dup1*, relative to *Dpse-polo* (Fig. 3a; Supplementary Table 3). Therefore, there is a consistent signal of faster amino acid evolution in *Dpse-polo-dup1*, yet the overall structure of Polo is conserved in both *Dpse-polo* and *Dpse-polo-dup1*.

Faster evolution of the *Dpse-polo-dup1* protein-coding sequence could be driven by relaxed purifying selection or stronger positive selection. To distinguish between these hypotheses, we used an MK test (McDonald and Kreitman 1991) to compare the amount of polymorphic and divergent synonymous and nonsynonymous changes within *D. pseudoobscura* and *D. miranda*. There was not a significant difference in the ratio of synonymous to nonsynonymous changes between polymorphic and divergent sites in *Dpse-polo* (Fig. 2b; *P* = 0.21 in Fisher's exact test). In contrast, there was a significant excess of nonsynonymous substitutions in *Dpse-polo-dup1* (Fig. 2c; *P* = 0.000003 in Fisher's exact test). An excess of nonsynonymous substitutions is a hallmark of positive selection, suggesting that the fast evolution of *Dpse-polo-dup1* was driven by adaptive substitutions.

### Male germline expression of Dpse-polo-dup1 increases fertility

To test if the rapid evolution of *Dpse-polo-dup1* has functional consequences, we used a GAL4>UAS system to express *Dpse-polo* and *Dpse-polo-dup1* in the *D. melanogaster* male germline. We also expressed an ovary-derived *polo* transcript (poloO) and a testis-derived *polo* transcript (poloT) from *D. melanogaster* (Supplementary Fig. 1) in the *D. melanogaster* male germline. We counted the number of male progeny and female progeny sired by each male expressing one of transcripts (Supplementary Table 4).

**Fig. 4.**
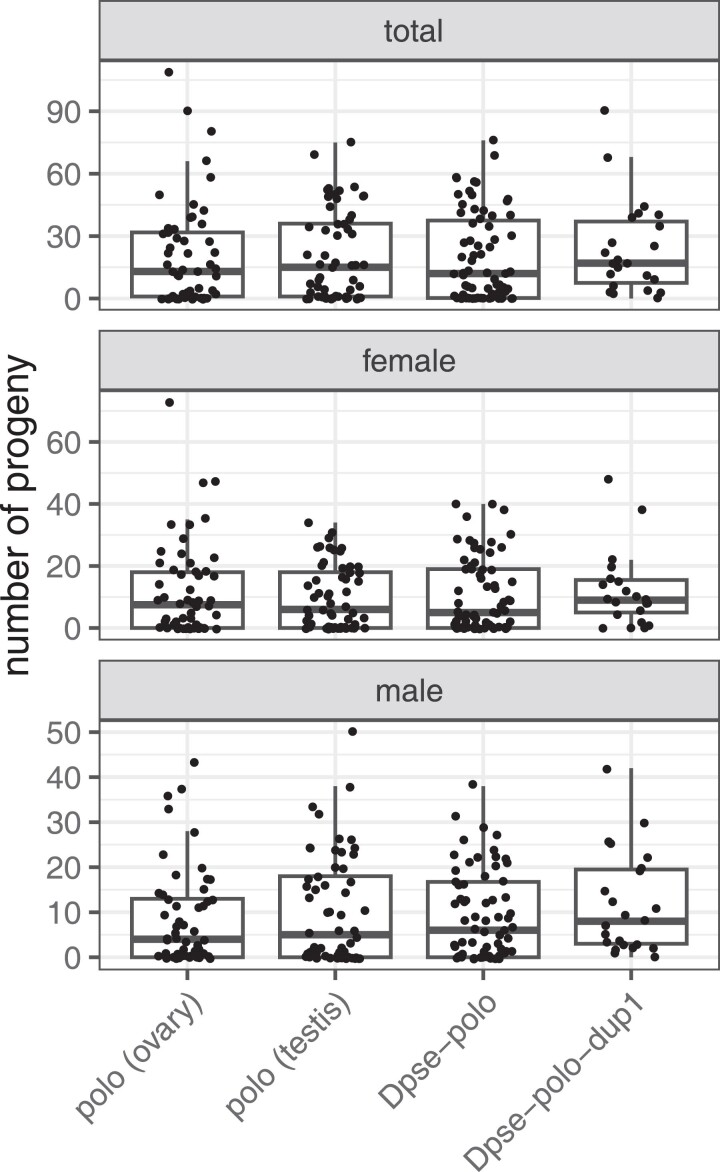
Number of progeny, number of female progeny, and number of male progeny sired by males with germline expression of different Plk transcripts. Males carried a transgene with a Plk transcript derived from *D. melanogaster* ovary mRNA [polo (ovary), i.e., poloO], a *D. melanogaster* testis mRNA [polo (testis), i.e., poloT], *Dpse-polo,* or *Dpse-polo-dup1*. Each dot shows the number of progeny sired by an individual male, and the box plots show the median and quartiles of the distribution for a given transgene.

We first tested whether expression of each Plk transcript in the *D. melanogaster* male germline affects the number of progeny sired. There was not a significant difference between the poloO and poloT transcripts on the total number of progeny, number of female progeny, or number of male progeny (all *P* > 0.39; Fig. 4). Similarly, there was not a significant difference between the effects of *Dpse-polo* and *Dpse-polo-dup1* on the number of total progeny, female progeny, or male progeny (all *P* > 0.24; Fig. 4).

We next tested if expressing the Plk transcripts in the *D. melanogaster* male germline affects if a male sires any progeny (i.e. whether a male sires 0 progeny or >0 progeny). Males that expressed *Dpse-polo-dup1* in their germline sired >0 progeny more frequently than males that expressed *Dpse-polo* (*z* = 1.818; *P* = 0.0691), poloO (*z* = −2.108; *P =* 0.0350), or poloT (*z* = −1.673; *P* = 0.0943). Approximately 20–25% of males that expressed *Dpse-polo*, poloT, or poloO sired zero progeny (Supplementary Table 4). In contrast, only one male (out of 22 or 4.3%) who expressed *Dpse-polo-dup1* in their germline sired zero progeny. There was not a significant difference in the number of males that sired zero progeny between those expressing *D. melanogaster* poloO and poloT in their germline (*z* = −1.037; *P* = 0.300). We observed similar effects when we only counted male or female progeny (Supplementary File 6).

### Male germline expression of ovary-derived Plk transcripts causes female-biased broods

**Fig. 5.**
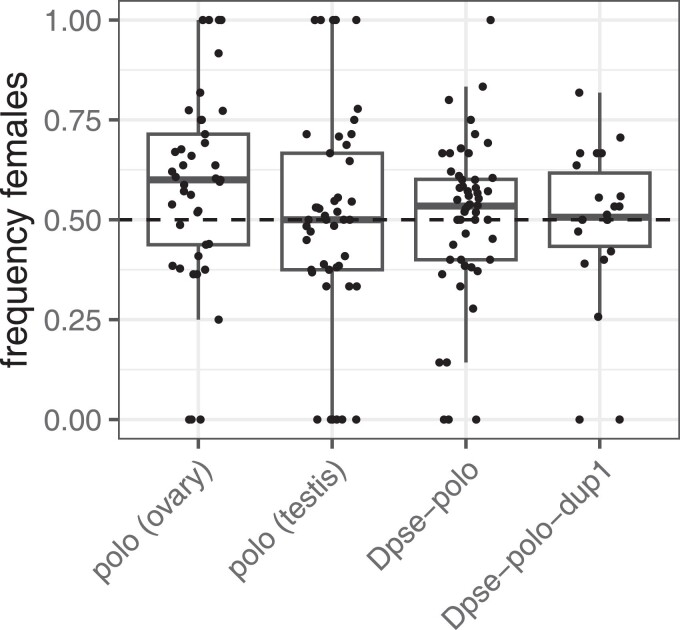
Frequency of female progeny sired by males with germline expression of different Plk transcripts. Males carried a transgene with a Plk transcript derived from *D. melanogaster* ovary mRNA [polo (ovary), i.e., poloO], a *D. melanogaster* testis mRNA [polo (testis), i.e., poloT], *Dpse-polo,* or *Dpse-polo-dup1*. Each dot shows the frequency of female progeny of progeny sired by an individual male [number of female progeny/(male + female progeny)], and the box plots show the median and quartiles of the distribution for a given transgene.

We also tested if expressing different Plk transcripts in the *D. melanogaster* male germline affects the ratio of female:male progeny sired. More female than male progeny were sired when we expressed poloO (*F*_1,46_ = 9.35, *P* = 0.0037) or *Dpse-polo* (*F*_1,61_ = 3.50, *P* = 0.066) in the male germline (Fig. 5). In contrast, there was not a significant difference in female and male progeny when we expressed poloT (*F*_1,49_ = 0.175, *P* = 0.68) or *Dpse-polo-dup1* (*F*_1,20_ = 0.0675, *P* = 0.80) in the male germline.

## Discussion

We showed that a duplicated Plk in the *D. pseudoobscura* genome (*Dpse-polo-dup1*) has testis-biased expression, while other *Drosophila* Plks are expressed more broadly in both males and females and in somatic and germline tissues (Figs. 1 and 2). We also demonstrated that *Dpse-polo-dup1* has the conserved structure of a canonical Plk, but its amino acid sequence evolved fast under positive selection (Fig. 3). Ectopic expression of *Dpse-polo-dup1* in the *D. melanogaster* male germline increased the probability of siring progeny relative to ectopic expression of other Plks (Fig. 4). In addition, expression of *Dpse-polo-dup1* in the *D. melanogaster* germline caused males to sire equal numbers of females and males, but male germline expression of other Plk transcripts resulted in an excess of female progeny (Fig. 5). Altogether, these results suggest that *Dpse-polo-dup1* is specialized for a male germline function because it does not have deleterious effects when expressed in the male germline. Alternatively, *Dpse-polo-dup1* may encode a hypomorphic Polo that does not decrease fertility or affect sex chromosome transmission, in contrast to other Plks.

### Gene duplication and testis specialization of meiotic genes

Our results suggest that the rapid evolution of *Dpse-polo-dup1* may be the result of adaptive fixations of amino acid substitutions that contribute to male germline specialization. Reis *et al*. (2011) hypothesized that Plk duplications with male-limited expression may accelerate male meiosis, which could provide a mechanism by which ectopic germline expression of *Dpse-polo-dup1* increases the fertility of *D. melanogaster* males. It is possible that the single copy *D. melanogaster polo* gene is constrained from germline-specific adaptation because of the diverse functions that Polo is required to perform. Plks are required for spindle attachment to and dissociation from the kinetochore in mitosis, female meiosis, and male meiosis (Sunkel and Glover 1988; Carmena *et al*. 1998; Herrmann *et al*. 1998; Archambault and Glover 2009; Das *et al*. 2016). There are functional differences among mitotic, female meiotic, and male meiotic spindles (Orr-Weaver 1995; Savoian and Glover 2014), which could create pleiotropic constraints opposing the specialization of *polo* function across mitotic and meiotic contexts (Wagner and Zhang 2011). In other words, improvements to Plk function in male meiosis could come at a cost to mitosis or female meiosis. Similar intersexual fitness tradeoffs have been documented in meiotic drive systems (Fishman and Saunders 2008). Duplication of *polo* in *D. pseudoobscura* may have allowed for the resolution of those pleiotropic conflicts via male meiotic specialization of *Dpse-polo-dup1* (Connallon and Clark 2011; Gallach and Betrán 2011; VanKuren and Long 2018; Hamada *et al*. 2020).

If male-specific subfunctionalization of a Plk duplication is advantageous, why does *D. melanogaster* not have subfunctionalized *polo* gene duplicates? The single *D. melanogaster polo* gene is autosomal (on chromosome *3L* or Muller element D), but *Dpse-polo* became *X*-linked when element D fused to the *X* chromosome, creating a neo-*X* chromosome. We hypothesize that the initial retention of *Dpse-polo-dup1* was favored after *Dpse-polo* became *X*-linked because *X* chromosome expression is reduced in the male germline (Vibranovski *et al*. 2009; Meiklejohn *et al*. 2011; Wei *et al*. 2024). Reduced *X* expression is thought to favor the retention of autosomal duplicates of *X*-linked genes when those genes are required for male meiosis or spermatogenesis (Betrán *et al*. 2002; Emerson *et al*. 2004; Marques *et al*. 2005; Potrzebowski *et al*. 2008; Meisel *et al*. 2009). The initial retention of *Dpse-polo-dup1* may have allowed for subsequent selection for male germline specialization, which resolved the antagonistic pleiotropy over meiotic and mitotic functions. This two-step process of selective retention of male germline-specific paralogs could explain why Plk duplications are not observed in other *Drosophila* species (Reis *et al*. 2011). More generally, this two-step process could explain the excess gene duplication from *Drosophila* neo-*X* chromosomes, and rapid (possibly adaptive) evolution of testis-expressed autosomal paralogs (Meisel *et al*. 2009, 2010).

Gene duplication may be a general way of resolving intersexual conflicts involving genes that encode meiotic proteins (Reis *et al*. 2011). For example, Plk genes have been duplicated and subfunctionalized in other taxa (Bettencourt-Dias *et al*. 2005; Habedanck *et al*. 2005), suggesting that duplication may be a common mechanism to resolve sexual conflict—or pleiotropic constraints more generally—imposed by differences in the spindle apparatus across mitosis and meiosis. In addition, the same autosome independently became a neo-*X* chromosome in *D. willistoni* as in *D. pseudoobscura*, and *mtrm* (a key interactor of *polo*) was similarly duplicated from the neo-*X* onto an autosome in *D. willistoni* (Xiang *et al*. 2007; Reis *et al*. 2011; Whitfield *et al*. 2013; Bonner *et al*. 2020). Furthermore, *mtrm* appears to have evolved under positive selection (Anderson *et al*. 2009), and there is evidence for divergence of Mtrm function in female meiosis across the *Drosophila* genus (Bonner and Hawley 2019). The evolutionary dynamics of *polo* and *mtrm* is therefore consistent with gene duplication resolving intersexual conflicts over sex differences in the meiotic spindle or kinetochore, a process that may be promoted by *X*-linkage of a gene required for both mitotis and meiosis.

### Mechanisms by which Plks could affect male meiosis and sex chromosome transmission

Our results are suggestive of mechanisms by which Plk transcripts could affect male fertility and progeny sex ratios. First, we hypothesize that the female-biased sex ratios observed when some Plks are expressed in the male germline are the result of an excess (>50%) of mature sperm carrying the *X* chromosome, relative to *Y*-bearing or nullo-*XY* sperm. An excess of *X-*bearing sperm would result in a female-biased sex ratio in the progeny because *X* chromosome dose determines sex in *Drosophila* (Erickson and Quintero 2007), i.e. zygotes with an *XX* genotype develop into females, and those with one *X* chromosome develop into males. We therefore hypothesize that male germline expression of Plk transcripts can affect the meiotic transmission of the sex chromosomes.

We observed that ectopic expression of poloO in the *D. melanogaster* male germline caused female-biased broods, while expression of poloT did not (Fig. 5). The poloO and poloT transgenes in our experiments had the same protein sequence, but they differed slightly in the UTRs they contained: poloT had a 5′-UTR that was 45 bp longer than poloO, while poloO had a 3′-UTR that was 17 bp longer than poloT (Supplementary Fig. 1; Supplementary Table 1). It is therefore possible that a region of the 5′-UTR found in poloT promotes equal transmission of the *X* and *Y* chromosomes or a region of the 3′-UTR found in poloO causes preferential transmission of the *X* chromosome (resulting in female-biased broods).

UTRs are known to affect both mitotic and meiotic functions of Plks. For example, there are two polyadenylation (pA) sites within the 3′-UTR of *D. melanogaster polo*, but the two transcripts encode the same protein (Fig. 1). The two *polo* mRNA products differ in their effects on kinetochore function, pupal metamorphosis, and female fertility, possibly because of differences in translational efficiencies between transcripts with different pA sites (Llamazares *et al*. 1991; Pinto *et al*. 2011; Oliveira *et al*. 2019). In addition, an allele in the human PLK1 3′-UTR affects mRNA secondary structure and stability (Akdeli *et al.* 2014), and shorter 3′-UTRs in many genes are associated with enhanced cell proliferation (Sandberg *et al*. 2008; Mayr and Bartel 2009). Some of these effects are caused by different pA sites, which should not differ between poloO and poloT—they share the same pA site that was engineered into their cloning backbone (Wang *et al*. 2012). However, the transcription rate of *polo* affects pA site selection, possibly via auto-regulatory feedback (Pinto *et al*. 2011). The GAL4>UAS system that we used may therefore have affected expression levels of *polo* transcripts in a way that shifted the relative usage of the pA site in the cloning backbone and a cryptic pA site in the 3′-UTR (Supplementary Fig. 1). It is also possible that the additional sequence in the poloO 3′-UTR may affect the testis function of *polo* via effects on transcript stability or translational efficiency. Additional experiments are required to test these hypotheses.

It is notable that a transcript that appears to have affected *X* chromosome transmission in spermatogenesis was cloned from the ovary (poloO), whereas a testis-derived transcript (poloT) had no such effects (Fig. 5). We cloned different transcripts from ovary and testis because the PCR primers that amplified *polo* transcripts in one tissue sample did not work in the other tissue sample. One explanation for our PCR results is that the testis and ovary transcripts of *polo* may have atypical UTR configurations (Fig. 1). Specifically, testis transcripts appear to have the longer 5′-UTR (similar to polo-RA) and the shorter 3′-UTR (similar to polo-RB). In contrast, ovary transcripts appear to have the shorter 5′-UTR (similar to polo-RB) and the longer 3′-UTR (similar to polo-RA). These different UTR configurations may explain why we could amplify a longer 5′-UTR in poloT and a longer 3′-UTR in poloO (Supplementary Fig. 1). These differences are also consistent with the hypothesis that a sequence in the 5′-UTR of *polo* has testis-beneficial effects or a sequence in the 3′-UTR is ovary beneficial. These testis- and/or ovary-specific effects may provide a mechanism for sexual conflict over transcript expression or splicing, possibly via transcript stability or translational efficiency.

A second important observation is that expression of *polo* or *Dpse-pol*o in the *D. melanogaster* male germline decreases male fertility relative to *Dpse-polo-dup1* (Fig. 4). We hypothesized that the higher relative fertility of males expressing *Dpse-polo-dup1* is caused by amino acid substitutions that optimized the protein for testis function, which is supported by the MK test for positive selection (Fig. 3). An alternative hypothesis is that high testis expression of Plks in the male germline decreases fertility, and *Dpse-polo-dup1* encodes a Plk with a mild loss of function causing a lower fertility cost. In the latter hypothesis, ectopic expression of *Dpse-polo-dup1* would be less costly than expression of the fully functional Plks encoded by *polo* and *Dpse-polo*. Negative effects of high *polo* expression have been shown in *D. melanogaster* intestinal stem cells, where constitutively active Polo suppresses intestinal stem cell proliferation, induces abnormal accumulation of β-tubulin in cells, and drives stem cell loss via apoptosis (Zhang *et al*. 2023). However, other experiments have shown Polo overexpression by 2.5-fold using GAL4>UAS does not affect its physiological function in mitosis (Martins *et al*. 2009). It therefore remains to be determined if *Dpse-polo-dup1* has fewer negative effects when ectopically expressed in the male germline, or if it has beneficial effects because of selection for testis specialization.

We hypothesize that differences in Plk transcript stability, translational efficiency, or protein-coding sequence affect chromosome segregation in male meiosis. This hypothesis is motivated by the observation that mutations to *polo* cause high rates of nondisjunction and sperm with abnormal DNA content (Sunkel and Glover 1988; Carmena *et al*. 1998, 2014; Herrmann *et al*. 1998). Polo may affect chromosomal transmission through its interactions with Mei-S332. Mei-S332 associates with centromeres in prometaphase of meiosis I, and phosphorylation by Polo is required for removal of Mei-S332 during segregation of sister chromatids in anaphase II (Goldstein 1981; Kerrebrock *et al*. 1992; Tang *et al*. 1998; Clarke *et al*. 2005). Mutation of *mei-S332* causes nondisjunction during meiosis II because of defective sister chromosome cohesion after metaphase I, which affects orientation going into meiosis II (Davis 1971; Goldstein 1980). Nondisjunction of autosomes could decrease fertility by increasing the frequency of autosomal aneuploidy. Another outcome of elevated meiosis II nondisjunction is that *mei-S332* mutant males produce an excess of *XX* sperm (i.e. coinheritance of sister chromatids), relative to *XY* sperm (coinheritance of homologous chromatids), in addition to an excess of nullo-*XY* sperm (Kerrebrock *et al*. 1992).

If ectopic expression of Plks in the male germline increases nondisjunction in meiosis II, this could provide insights into the mechanisms that affect both male fertility and sex ratios (Figs. 4 and 5). As described above, nondisjunction of autosomes in meiosis II would result in aneuploid progeny. Autosomal aneuploids are inviable, which could explain the decreased fertility of males expressing some Plk transcripts. However, nondisjunction of the sex chromosomes in meiosis II could be expected to produce an excess of sperm that can give rise to male progeny. An excess of males would be expected because nondisjunction of the *X* chromosome in meiosis II produces *XX* and nullo-*XY* sperm, which will result in female (*XXX*) and sterile male (*XO*) zygotes, respectively, upon fertilization. Notably, *XXX* females have dramatically reduced viability (Lindsley and Zimm 1992), suggesting that *X* nondisjunction should create male-biased progeny. In addition, nondisjunction of the *Y* chromosome in meiosis II produces *YY* and nullo-*XY* sperm, which will only result in male zygotes (*XYY* or *XO*). We therefore would expect male-biased sex ratios if there were a high rate of sex chromosome nondisjunction in meiosis II in the male germline. In contrast, we observed female-biased sex ratios when some Plk transcripts were expressed in the male germline (Fig. 5), suggesting that nondisjunction alone cannot explain the effects of Plk expression on *X* chromosome transmission.

### Sex ratio distortion and sexual conflict

The mechanisms responsible for naturally occurring sex ratio drive may shed light on why we observed female-biased broods upon expression of some Plks in the male germline (Fig. 5). Female- or male-biased sex ratios can arise via meiotic drive or segregation distortion, and the mechanisms by which this occurs differ across species and between oogenesis and spermatogenesis (Lindholm *et al*. 2016; Courret *et al*. 2019). Most relevant to our results is the *D. simulans* Paris system, where an *X*-linked allele causes female-biased sex ratios when males carry the driving *X* chromosome (Cazemajor *et al*. 1997). The driving *X* increases the frequency of nondisjunction of *Y* chromatids in meiosis II, resulting in nulo-*XY* and *YY* sperm, but the *YY* sperm fail to mature (Cazemajor *et al*. 2000). A similar phenomenon may occur in the *D. pseudoobscura* sex ratio drive system, where an *X*-linked allele causes a reduction in the frequency of *Y*-bearing spermatocytes (Novitski *et al*. 1965; Policansky and Ellison 1970). Therefore, while sex chromosome nondisjunction on its own may be predicted to cause male-biased sex ratios, there is precedent for female-bias if *Y* nondisjunction in meiosis II fails to produce viable gametes.

The *D. simulans* Paris drive system is caused by an allele on the driving *X* chromosome in a gene that encodes a heterochromatin protein which fails to package *Y* heterochromatin properly for meiosis (Helleu *et al*. 2016). While Plks have not been directly implicated in the regulation of heterochromatin, they do have important interactions with heterochromatic regions of chromosomes during meiosis. Specifically, Polo interacts with proteins, such as MEI-S332, that are essential for centromere cohesion during meiosis (Clarke *et al*. 2005), and centromeres are enriched for constitutive heterochromatin (Mteirek *et al*. 2014). Future work should explore if *Drosophila* Plks affect meiotic (in particular, *Y* chromosome) heterochromatin, which could explain the female-biased sex ratios we observed. In addition, occasionally *XO* males are sired by *D. simulans* fathers carrying the Paris *X* chromosome, suggesting that some nullo-*XY* sperm are produced (Cazemajor *et al*. 2000). Therefore, the hypothesis that ectopic Plk expression increases the rate of *Y* chromosome nondisjunction could be further tested by assaying the genotypes of male progeny.

Another similarity between our results and previously documented segregation distortion systems is that most genes that cause segregation distortion in *Drosophila* are recent gene duplications that acquired germline-specific expression (Merrill *et al*. 1999; Montchamp-Moreau *et al*. 2006; Tao *et al*. 2007a, 2007b; Helleu *et al*. 2016; Lin *et al*. 2018). For example, the *D. melanogaster Segregation Distorter* (*SD*) chromosome is preferentially transmitted relative to wild-type second chromosomes in *SD/+* heterozygous males (Temin *et al*. 1991; Larracuente and Presgraves 2012). The *Sd* locus that is responsible for *SD* drive is a truncated duplication of a gene encoding the Ran GTPase-activating protein (*RanGAP*), and the *Sd* gene is sufficient to create the driving effect of the *SD* chromosome (Merrill *et al*. 1999). In addition, simply overexpressing *RanGAP* in the male germline causes segregation distortion in a way that mimics the effect of the *SD* locus (Kusano *et al*. 2001). This driving effect of overexpression is reminiscent of the sex ratio distortion we observe when ectopically expressing poloO or *Dpse-polo* in the male germline (Fig. 5).

Sex ratio distortion and meiotic drive are often framed as intragenomic conflicts, which are usually studied independently of intralocus sexual antagonism (Lindholm *et al*. 2016). Our results provide evidence that Plk expression can create intragenomic conflict and, more specifically, sexually antagonistic effects (Rowe *et al*. 2018). We hypothesize that *polo* alleles that optimize function in mitotic or female meiotic chromosome segregation can have deleterious effects when expressed during male meiosis. We observe these effects when we express *polo* or *Dpse-polo* in the *D. melanogaster* male germline, and female-biased broods are sired. In contrast, we hypothesize that selection to optimize *Dpse-polo-dup1* for male germline function ameliorates those deleterious effects, consistent with a model in which gene duplication has resolved a sexual conflict (Connallon and Clark 2011; Gallach and Betrán 2011). This hypothesis explains why ectopic expression of *Dpse-polo-dup1* in the *D. melanogaster* male germline increases fertility (relative to *polo* and *Dpse-polo*) and does not skew sex ratios. This relationship between fertility and sex ratios is consistent with previous work that has identified fertility costs associated with meiotic drive (Zanders and Unckless 2019). Our results therefore provide a link between intralocus sexual antagonism and sex ratio drive, but it is not clear if sexual conflicts over meiotic functions respond to or cause sex ratio drive.

## Conclusions

We showed that a fast evolving, testis-expressed Plk duplication in the *D. pseudoobscura* genome (*Dpse-polo-dup1*) did not impose fertility costs nor did it skew progeny sex ratios when expressed in the *D. melanogaster* male germline. In contrast, ectopic testis expression of ovary-derived Plk transcripts caused males to sire female-biased broods. These results are consistent with adaptive specialization of *Dpse-polo-dup1* for male germline-specific function, possibly related to unique requirements associated with the male meiotic spindle apparatus. A similar testis specialization could explain alternative UTRs between ovary and testis-expressed *polo* transcripts in *D. melanogaster*. Alternatively, *Dpse-polo-dup1* may be a hypomorphic Plk variant that does not have deleterious effects when overexpressed in the male germline, in contrast to other Plks. Initially *Dpse-polo-dup1* may have been selectively retained because neo-*X*-linkage caused decreased male germline expression of the ancestral *Dpse-polo* locus, favoring an autosomal paralog to compensate. This could explain why *D. pseudoobscura* has a testis-expressed Plk paralog, but *D. melanogaster* does not. These results more generally provide evidence for divergent selection pressures on spindle assembly genes in mitosis, female meiosis, and male meiosis. One consequence of these divergent selection pressures may be that different Plks vary in their effects on nondisjunction during meiosis II in males. We hypothesize that these divergent selection pressures create pleiotropic conflicts or sexual antagonism, which can then be resolved by duplication and germline specialization of a paralog.

## Supplementary Material

jkae273_Supplementary_Data

## Data Availability

Strains used in this experiment are available upon request. The authors affirm that all data necessary for confirming the conclusions of the article are present within the article, figures, tables, and Supplementary material. Supplementary material includes PCR primers to amplify Plk sequences and transgenic strains created that carry transcripts; results of statistical analyses; RNA-seq data and code to analyze the data; coding sequence alignments; and data from fly experiments with code to analyze the data. Supplemental material available at G3 online.
