# Supplementary material for: Testis- and ovary-expressed polo-like kinase transcripts and gene duplications affect male fertility when expressed in the Drosophila melanogaster germline
Source: G3 (Bethesda). 2024 Nov 20;15(1):jkae273. doi: 10.1093/g3journal/jkae273 (PMC11708218; doi:10.1093/g3journal/jkae273)
Supplement: jkae273_Supplementary_Data [file jkae273_supplementary_data.zip › Supplemental_Figure_S1_G3-2024-405417.pdf]

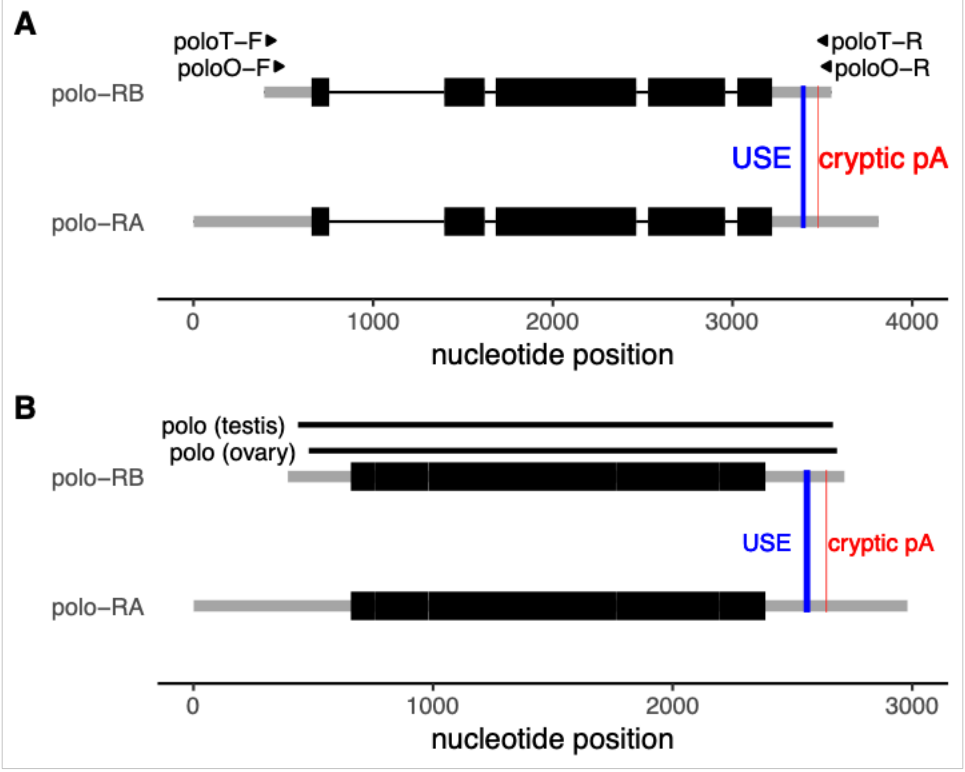

Supplemental Figure S1. *D. melanogaster polo* is spliced into two different isoforms that differ in their untranslated regions (UTRs). The location of a conserved upstream sequence element (USE) that affects usage of the polo-RB polyadenylation (pA) site is shown by a blue line (Oliveira *et al.* 2019). A cryptic pA signal, used when the USE is mutated, is shown by a red line. **A.** The *polo* gene region is diagrammed, with two alternative transcripts of *polo* shown (polo-RA and polo-RB). Thick black boxes show protein coding exons. Medium gray bars show the 5'-UTR (left) and 3'-UTR (right). Introns are shown as thin black lines. The location of forward (F) and reverse (R) primers used to clone cDNA from testis (poloT) and ovary (poloO) transcripts are shown. **B.** The mRNA of the alternative *polo* transcripts are diagrammed. The regions cloned from testis and ovary are shown above the polo-RB transcript.
