# Supplementary material for: Testis- and ovary-expressed polo-like kinase transcripts and gene duplications affect male fertility when expressed in the Drosophila melanogaster germline
Source: G3 (Bethesda). 2024 Nov 20;15(1):jkae273. doi: 10.1093/g3journal/jkae273 (PMC11708218; doi:10.1093/g3journal/jkae273)
Supplement: jkae273_Supplementary_Data [file jkae273_supplementary_data.zip › Supplemental_File_S2_G3-2024-405417.pdf]

RNA-seq Data Analysis

2024-04-05

Load data and setup initial objects

Load the *Drosophila pseudoobscura* RNA-seq data:

```
DpseNRCfb <- read.delim("SupFileS01_dpse.nrc.FB.txt")
```

The following sample types are present in these data:

sample_ID	sample_type
ac	abdomen without digestive or reproductive system
dg	digestive plus excretory system
go	gonad
hd	head
re	reproductive system without gonad
tx	thorax without digestive system
wb	whole body

Male (`m`) and female (`f`) samples were collected for each sample type. There are four replicates per sample type. Calculate the `median` expression value across all replicates for each sex by sample type combination:

```
DpseAvg <- data.frame(
    FBgnID = DpseNRCfb$FBgnID,
    dpse_f_ac = apply(DpseNRCfb[, 2:5 ],1, median),
    dpse_f_dg = apply(DpseNRCfb[, 6:9 ],1, median),
    dpse_f_go = apply(DpseNRCfb[,10:13],1, median),
    dpse_f_hd = apply(DpseNRCfb[,14:17],1, median),
    dpse_f_re = apply(DpseNRCfb[,18:21],1, median),
    dpse_f_tx = apply(DpseNRCfb[,22:25],1, median),
    dpse_f_wb = apply(DpseNRCfb[,26:29],1, median),
    dpse_m_ac = apply(DpseNRCfb[,30:33],1, median),
    dpse_m_dg = apply(DpseNRCfb[,34:37],1, median),
    dpse_m_go = apply(DpseNRCfb[,38:41],1, median),
    dpse_m_hd = apply(DpseNRCfb[,42:45],1, median),
    dpse_m_re = apply(DpseNRCfb[,46:49],1, median),
    dpse_m_tx = apply(DpseNRCfb[,50:53],1, median),
    dpse_m_wb = apply(DpseNRCfb[,54:57],1, median)
)
```

The two *D. pseudoobscura polo* genes (*Dpse-polo* and *Dpse-polo-dup1*) have the following annotation IDs:

	Dpse_polo	Dpse_polo_dup1
ID	GA11545	GA25172
transcript	XM_001353282.5	XM_002132425.3
geneID	LOC4812761	LOC6903594
protein	XP_001353318.5	XP_002132461.2
FlyBaseID	FBgn0071596	FBgn0246554

Compare expression of D. pseudoobscura polo genes versus genome-wide distribution

Generate a ridgeplot comparing *Dpse-polo* and *Dpse-polo-dup1* expression against the distribution of expression in each sex by sample type combination:

```
library(ggplot2)
library(ggridges)
library(cowplot)
library(grid)

data.framer <- function(FBIDs, Sex, Tissue, Expr){
    data.frame(
        FBgnID = FBIDs,
        sex = Sex,
        tissue = Tissue,
        express = Expr
    )
}
DpseAvg.df <- rbind(
    data.framer(DpseAvg$FBgnID, "female","abdomen", DpseAvg$dpse_f_ac),
    data.framer(DpseAvg$FBgnID, "female","digestive", DpseAvg$dpse_f_dg),
    data.framer(DpseAvg$FBgnID, "female"," gonad", DpseAvg$dpse_f_go),
    data.framer(DpseAvg$FBgnID, "female","head", DpseAvg$dpse_f_hd),
    data.framer(DpseAvg$FBgnID, "female"," reproductive", DpseAvg$dpse_f_re),
    data.framer(DpseAvg$FBgnID, "female","thorax", DpseAvg$dpse_f_tx),
    data.framer(DpseAvg$FBgnID, "female","whole body", DpseAvg$dpse_f_wb),
    data.framer(DpseAvg$FBgnID, "male","abdomen", DpseAvg$dpse_m_ac),
    data.framer(DpseAvg$FBgnID, "male","digestive", DpseAvg$dpse_m_dg),
    data.framer(DpseAvg$FBgnID, "male"," gonad", DpseAvg$dpse_m_go),
    data.framer(DpseAvg$FBgnID, "male","head", DpseAvg$dpse_m_hd),
    data.framer(DpseAvg$FBgnID, "male"," reproductive", DpseAvg$dpse_m_re),
    data.framer(DpseAvg$FBgnID, "male","thorax", DpseAvg$dpse_m_tx),
    data.framer(DpseAvg$FBgnID, "male","whole body", DpseAvg$dpse_m_wb)
)

DpseRNAseq <- ggplot(DpseAvg.df, aes(y=tissue, x=log10(express+1), fill=sex, color=sex)) +
    geom_density_ridges(scale=0.9) +
    scale_color_manual(values=c("female"="red", "male"="darkblue")) +
    scale_fill_manual(values=c("female"="pink", "male"="lightblue")) +
    geom_point(data=subset(DpseAvg.df, FBgnID=="FBgn0071596"),
               color="black", fill="black", shape=1, size=3) +
    geom_point(data=subset(DpseAvg.df, FBgnID=="FBgn0246554"),
               color="black", fill="black", shape=2, size=3) +
```

```
    scale_x_continuous(expression(log[10]~"(normalized"~expression~"level)"),
                       limits=c(0, 6)) +
    scale_y_discrete("") +
    facet_wrap(~sex, ncol=2) +
    theme_bw() +
    theme(legend.position = "none")

ggdraw(DpseRNAseq) +
  draw_label("\u25cb", size=11, x=0.84, y=0.9) +
  draw_label("\u25b3", size=11, x=0.84, y=0.87) +
  draw_label("Dpse-polo", size=8, x=0.85, y=0.9, hjust=0) +
  draw_label("Dpse-polo-dup1", size=8, x=0.85, y=0.87, hjust=0)
```

```
## Picking joint bandwidth of 0.145
```

```
## Picking joint bandwidth of 0.138
```

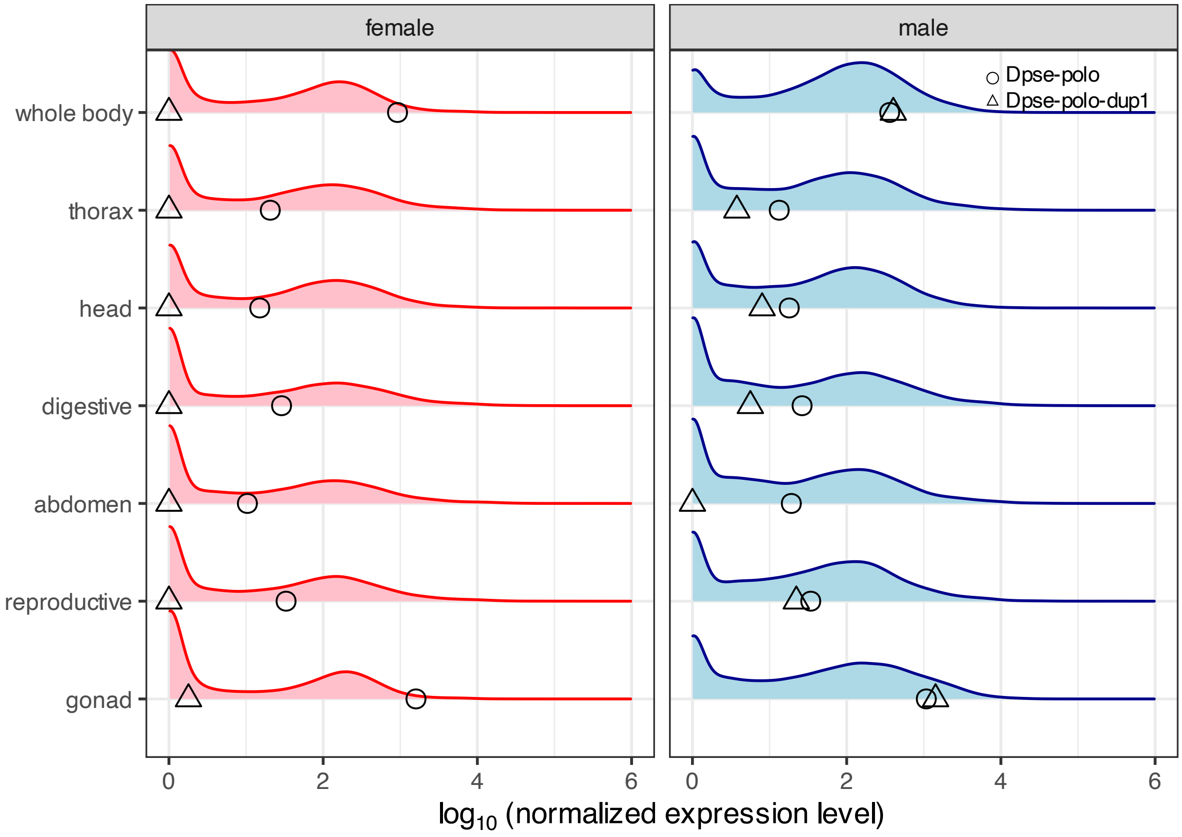

Compare tissue-specificity (tau) of D. pseudoobscura polo genes against genome

Calculate tau using the equation:

$$\tau = \sum_{i=1}^{N} \frac{1 - \frac{\log S_i}{\log S_{max}}}{N-1}$$

For our data, N = 6 (see tissue list above). We will define a function to calculate tau:

```
tau.calc <- function(datas){
    N <- length(datas)
    Smax <- max(datas)
    taus <- 1 - log10(datas+1)/log10(Smax+1)
    sum(taus)/(N-1)
}
DpseAvg$tau_female <- apply(
  subset(DpseAvg, select=c(dpse_f_ac, dpse_f_dg, dpse_f_go, dpse_f_hd, dpse_f_re, dpse_f_tx)),
  1, tau.calc)
DpseAvg$tau_male <- apply(
  subset(DpseAvg, select=c(dpse_m_ac, dpse_m_dg, dpse_m_go, dpse_m_hd, dpse_m_re, dpse_m_tx)),
  1, tau.calc)
```

Prepare object to be used to make graphs showing tau values and plot:

```
YaxisSpacer = "                      "
DpseAvgTau <- na.omit(rbind(
    data.frame(gene=DpseAvg$FBgnID, tau=DpseAvg$tau_female, sex="female", Yaxis=YaxisSpacer),
    data.frame(gene=DpseAvg$FBgnID, tau=DpseAvg$tau_male, sex="male", Yaxis=YaxisSpacer)))

DpseTau <- ggplot(DpseAvgTau, aes(y=Yaxis, x=tau, fill=sex, color=sex)) +
    geom_density_ridges(scale=0.9, quantile_lines = TRUE, quantiles = 2) +
    scale_color_manual(values=c("female"="red", "male"="darkblue")) +
    scale_fill_manual(values=c("female"="pink", "male"="lightblue")) +
    geom_point(data=subset(DpseAvgTau, gene=="FBgn0071596"),
               color="black", fill="black", shape=1, size=3) +
    geom_point(data=subset(DpseAvgTau, gene=="FBgn0246554"),
               color="black", fill="black", shape=2, size=3) +
    scale_x_continuous(expression("expression"~breadth~"("~tau~")"), limits=c(0,1)) +
    scale_y_discrete("") +
    facet_wrap(~sex, ncol=2) +
    theme_bw() +
    theme(legend.position = "none")

ggdraw(DpseTau) +
  draw_label("\u25cb", size=11, x=0.84, y=0.9) +
  draw_label("\u25b3", size=11, x=0.84, y=0.87) +
  draw_label("Dpse-polo", size=8, x=0.85, y=0.9, hjust=0) +
  draw_label("Dpse-polo-dup1", size=8, x=0.85, y=0.87, hjust=0)
```

```
## Picking joint bandwidth of 0.0441

## Picking joint bandwidth of 0.0417
```

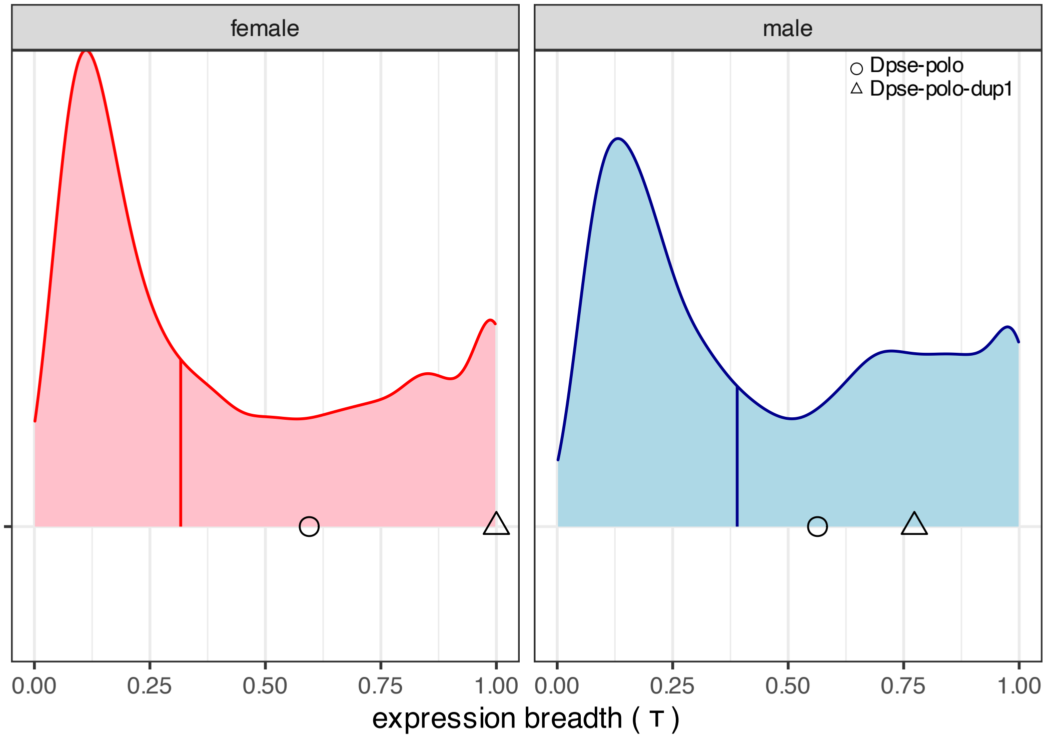
female
male
Dpse-polo
Dpse-polo-dup1
0.00
0.25
0.50
0.75
1.00
expression breadth (τ)
